# Mechanism study of exercise intervention on circadian disruption in Alzheimer’s disease

**DOI:** 10.3389/fnins.2025.1696673

**Published:** 2025-12-11

**Authors:** Mingzheng Zhang, Lei Shi, Xiangqi Meng, Yahong Dong, Yinjie Sun, Qiheng Qian, Qiguan Jin

**Affiliations:** 1 College of Physical Education, Yangzhou University, Yangzhou, Jiangsu, China; 2 Suzhou Hospital of Traditional Chinese Medicine, Suzhou, Jiangsu, China

**Keywords:** Alzheimer’s disease, circadian rhythm disruption, exercise intervention, Aβ clearance, melatonin rhythm, core clock genes

## Abstract

Alzheimer’s disease (AD) induces profound circadian disruption—characterized by suprachiasmatic nucleus (SCN) degeneration, Aβ and tau pathology, and aberrant melatonin secretion which results in fragmented sleep–wake cycles and cognitive decline. Emerging evidence indicates that exercise significantly ameliorates these circadian rhythm and sleep–wake disturbances through multiple mechanisms. Specifically, exercise accelerates the removal of Aβ and tau by activating autophagy–lysosomal degradation and glymphatic clearance, thereby reducing the neuropathological burden. Furthermore, exercise also upregulates neuroprotective pathways and strengthens the amplitude and phase stability of core clock gene oscillations, which in turn restores robust melatonin rhythmicity and SCN function. Collectively, these effects break the vicious cycle between AD pathology and circadian disruption, stabilizing circadian homeostasis and improving cognitive function. These mechanistic insights highlight exercise as a robust non-pharmacological chronotherapeutic strategy for ameliorating circadian disruption in AD. These insights support the development of personalized, chronotype-tailored exercise interventions to resynchronize circadian rhythms and ultimately improve sleep quality and cognitive function in patients with AD.

## 1 Introduction

Currently, over 47 million people worldwide have dementia, and this number is projected to rise to approximately 90 million by 2030 (Van Erum et al., 2018). Among all dementias, Alzheimer’s disease represents the predominant etiology, comprising about 75% of cases (Van Erum et al., 2018). Approximately 25–60% of AD patients exhibit circadian rhythm disturbances, with over 80% of those older than 65 years showing significant circadian disruption (Blazer et al., 1995; Van Someren, 2000). In AD patients, degeneration of SCN neurons, disruption of melatonin rhythms, and synergistic effects of *β*-amyloid (Aβ)/Tau pathology and neuroinflammation result in a fragmented sleep–wake cycle, diminished amplitude, and phase misalignment, which have profound adverse effects on cognitive function.

Circadian rhythm refers to the endogenous, approximately 24 h oscillation in physiology and behavior that persists without external cues and is synchronized to the environment by zeitgebers such as light, activity, and feeding (Shearman et al., 2000). At the cellular level it arises from transcription–translation feedback loops coordinated by the suprachiasmatic nucleus (SCN). In the core loop, CLOCK and BMAL1 heterodimerize, bind E-box elements, and activate transcription of Period (Per1/2/3) and Cryptochrome (Cry1/2); PER and CRY proteins then accumulate, re-enter the nucleus, and inhibit CLOCK: BMAL1, thereby closing the negative feedback cycle (Ko and Takahashi, 2006). An auxiliary loop involving RORα/*β*/*γ* and REV-ERBα/β acts on ROREs in the BMAL1 promoter to set phase and amplitude. Post-translational modifiers such as casein kinase 1δ/*ε* tune periodicity, and SCN coupling aligns cellular clocks across tissues, producing coherent daily programs of gene expression, metabolism, hormone secretion, and rest–activity behavior (Kondratova and Kondratov, 2012).

Circadian rhythm disruption denotes a state in which the internal circadian system is impaired or misaligned with external timing, either because intrinsic clock mechanisms are damaged or because entrainment to environmental cues fails (Meyer et al., 2022; Auger et al., 2015). Clinically, this manifests as difficulty initiating or maintaining sleep, reduced nighttime sleep quality, excessive daytime sleepiness, multiple daytime sleep episodes, loss of a clear day–night boundary, impaired deep sleep at night, and frequent napping during the day (Sun and Chen, 2022).

In AD, Aβ plaques and tau neurofibrillary tangles are increasingly recognized to be intimately linked with core clock gene dysregulation and corresponding alterations in melatonin signaling. Disruptions in clock gene function and diminished melatonin production in AD can impair the brain’s capacity to clear toxic proteins such as Aβ and tau, whereas accumulating Aβ in turn further disrupts circadian regulators and suppresses melatonin, creating a self-perpetuating cycle. This bidirectional interplay suggests that Aβ/tau pathology and circadian disruption should not be viewed as isolated phenomena but rather as interconnected elements of AD pathophysiology. Accordingly, there is growing interest in holistic therapeutic strategies that simultaneously target these mechanisms. One promising example is exercise—a readily accessible chronotherapeutic intervention—which has been shown to realign circadian clock gene oscillations and restore melatonin rhythmicity while concurrently promoting the clearance of Aβ and tau (Hu et al., 2025). However, research on exercise and circadian disruption in Alzheimer’s disease has largely focused on observed effects, while the underlying mechanisms remain insufficiently explored.

Animal studies have shown that scheduled voluntary aerobic exercise can remodel circadian rhythms and improve circadian disruption in mice (Hughes et al., 2021). Human trials indicate that time-based personalized aerobic exercise prescriptions can alleviate circadian disruption in healthy young adults, demonstrating the efficacy of aerobic exercise in ameliorating circadian disturbances in healthy organisms (Thomas et al., 2020). Therefore, whether exercise can modulate circadian disruption in AD patients—and by what mechanisms—is of great interest. Studies show that circadian disruption in patients with AD differs from that in normally aging individuals, likely due to Aβ and tau pathology, clock genes, and melatonin secretion (Eckhardt et al., 2025). Exercise interventions may improve AD-related circadian disruption through multiple molecular mechanisms—such as promoting Aβ clearance, mitigating Tau pathology, enhancing clock-gene expression and melatonin secretion, and modulating brain network function. This review focuses on: (1) the interplay between typical AD pathology and the circadian system; (2) exercise-mediated regulation of clock genes; and (3) mechanisms by which targeted melatonin modulation via exercise impacts circadian homeostasis in AD patients.

## 2 AD pathological hallmarks and circadian disruption

### 2.1 Aβ pathology and circadian rhythms

AD is an age-related neurodegenerative disease, with β-amyloid (Aβ) plaques and Tau protein aggregates as its cardinal features (Scheltens et al., 2021; Nigam et al., 2017; Holth et al., 2019). Sleep plays a crucial role in Aβ clearance via the glymphatic system: during wakefulness, Aβ production and release into the extracellular space increase, whereas sleep enhances Aβ removal; circadian disruption leads to Aβ accumulation in the brain, ultimately causing neuronal death (Scheltens et al., 2021; van Hattem et al., 2024). Aβ deposition further disrupts the sleep–wake cycle, increasing wakefulness and exacerbating circadian disruption (Roh et al., 2012; Xie et al., 2013). Even reduced sleep duration alone elevates Aβ levels (Winer et al., 2021). Animal experiments demonstrate that in APP/PS1 transgenic mice, Aβ plaque deposition in the thalamic reticular nucleus (TRN) leads to local neuronal hyperexcitability and inhibits sleep spindle generation, thereby fragmenting sleep (Katsuki et al., 2022). Meanwhile, experimental evidence indicates that Aβ-induced degradation of BMAL1 and CBP precipitates circadian rhythm disruption in Alzheimer’s disease. Abnormal Aβ accumulation within the thalamic reticular nucleus (TRN) and the basal forebrain—both critical hubs for slow-wave sleep (SWS) generation—directly impairs regional network function, leading to SWS reduction and exacerbating circadian disruption (Wang and Holtzman, 2020). Mechanistically, Aβ promotes proteasomal degradation of key circadian regulators: the transcriptional co-activator CBP (CREB-binding protein) and the core transcription factor BMAL1, which normally heterodimerizes with CLOCK. Loss of the BMAL1–CBP complex diminishes its binding affinity at the Per2 promoter, thereby attenuating rhythmic Per2 mRNA oscillations and PER2 protein expression. This cascade destabilizes circadian output signals, which in turn accelerates Aβ deposition, establishing a vicious cycle of circadian disruption and amyloid pathology (Lucey et al., 2018; Song et al., 2015).

### 2.2 Tau pathology and circadian rhythms

Hyperphosphorylated Tau protein, forming neurofibrillary tangles, is not only a hallmark of AD pathology but also profoundly affects sleep–wake system stability (Holth et al., 2019). In Tau pathology mouse models, p-Tau immunoreactivity in the SCN region is accompanied by a significant reduction in the oscillation amplitude of core clock genes BMAL1 and Per2, directly impairing the molecular feedback loop of the central clock (Han et al., 2022). Tau aggregation leads to SCN neuronal dysfunction and loss of network synchrony, reducing SCN responsiveness to zeitgebers; this manifests as attenuated circadian amplitude, phase disorganization, and even complete loss of rhythmic behavior (Son et al., 2024). Moreover, Tau pathology activates microglia and astrocyte–mediated neuroinflammation, releasing pro-inflammatory cytokines that further inhibit CLOCK/BMAL1 transcriptional activity, disrupt the Per/Cry negative feedback loop, desynchronize clock gene expression timing, reduce nighttime melatonin peak amplitude and duration, and exacerbate disrupted sleep architecture and circadian flattening (Yang et al., 2025). In summary, hyperphosphorylated Tau impairs SCN function and disrupts circadian homeostasis in AD.

## 3 Exercise-mediated modulation of AD pathology to improve circadian disruption

### 3.1 Exercise accelerates Aβ clearance

Aerobic exercise has been shown to directly accelerate Aβ clearance via multiple mechanisms, thereby interrupting the vicious cycle between Aβ pathology and circadian rhythm disruption (Tan et al., 2021). Principal Aβ clearance pathways include autophagy, non-amyloid metabolic routes, lipid raft remodeling, and SIRT1-mediated deacetylation.

#### 3.1.1 Autophagy–lysosome pathway

Autophagy is a cell-intrinsic degradation process that removes damaged organelles and invading pathogens to maintain intracellular homeostasis (Nixon and Rubinsztein, 2024). It is a critical route for intracellular Aβ clearance, aerobic exercise activates the autophagy–lysosome pathway (e.g., reducing p62 levels), enhances Aβ degradation efficiency, effectively inhibits mTOR hyperactivation, restores autophagic flux, and counteracts Aβ accumulation due to autophagic blockade in AD. A 12-week treadmill regimen effectively reduced Aβ deposition in APP/PS1 mice by enhancing autophagy–lysosome activity (Zhao et al., 2018). Wenfeng Liu et al. found that 12 weeks of aerobic exercise activated the AdipoR1/AMPK/TFEB pathway in AD rats, bolstered lysosomal function, mitigated aberrant autophagy, and thereby reduced Aβ accumulation (Jian et al., 2022).

#### 3.1.2 PGC-1α/FNDC5/BDNF axis

The proteolytic conversion of amyloid precursor protein (APP) to A*β* involves sequential cleavage by β-secretase (BACE1), which generates the C-terminal fragment C99, followed by *γ*-secretase–mediated processing of C99 to release A*β* peptides (Zhang and Song, 2013). Exercise induces expression of the myokine FNDC5 via a PGC-1α–dependent pathway, directly inhibiting BACE1-mediated β-site cleavage of APP and reducing Aβ generation; FNDC5 also upregulates hippocampal BDNF, indirectly enhancing *α*-secretase activity. This PGC-1α → FNDC5 → BDNF cascade forms an anti-Aβ network. Research has shown that hippocampal PGC-1α and BDNF protein levels are significantly lower in Aβ model rats than in controls. Moderate treadmill exercise, by augmenting AMPK activity, significantly increased hippocampal PGC-1α and BDNF levels in Aβ model rats by 48 and 47%, respectively (Azimi et al., 2018).

#### 3.1.3 Lipid raft remodeling

Lipid rafts are cholesterol- and sphingolipid-enriched plasma-membrane microdomains that concentrate BACE1 and *γ*-secretase, integrate transmembrane signaling, and play a central role in receptor regulation and cellular homeostasis. In Alzheimer’s disease (AD), rafts serve as pathological platforms for aberrant A*β* metabolism: by clustering β- and *γ*-secretases, they enhance amyloidogenic APP cleavage, and their dynamic reorganization disrupts autophagic–lysosomal degradation, impeding Aβ clearance and accelerating neurodegeneration (Cordy et al., 2006; Zhang et al., 2019). Exercise has been shown to attenuate both the biogenesis and stability of lipid rafts via multiple convergent mechanisms. Firstly, chronic aerobic training upregulates cholesterol-24-hydroxylase (CYP46A1), reducing neuronal cholesterol content and limiting the substrate pool required for raft assembly. Secondly, exercise downregulates the scaffold proteins caveolin-1 and flotillin-1, which are essential for raft nucleation and maturation, thereby diminishing the coalescence of APP-processing enzymes within these domains. Thirdly, enhanced AMPK activity during exercise phosphorylates and inhibits acetyl-CoA carboxylase, shifting membrane lipid composition away from saturated fatty acids that favor raft rigidity. Collectively, these changes disrupt the spatial colocalization of BACE1 and *γ*-secretase, preventing efficient *β*- and γ-site APP cleavage. By dispersing amyloidogenic enzyme complexes into non-raft regions—where APP predominantly encounters *α*-secretase—exercise not only suppresses Aβ production but, through concomitant improvements in autophagic–lysosomal function, also promotes the degradation of residual peptide (Zeng et al., 2020).

#### 3.1.4 SIRT1-dependent pathway

The SIRT1 pathway is implicated in multiple neurodegenerative processes. In AD, SIRT1 is downregulated, resulting in increased Aβ production, whereas SIRT1 overexpression can reverse this pathology, highlighting SIRT1’s importance in Aβ regulation. Aerobic exercise-induced SIRT1 upregulation activates ADAM10 via deacetylation, enhancing non-amyloidogenic metabolism; SIRT1 also upregulates PGC-1α to inhibit BACE1 expression, and, in concert with retinoic acid receptor *β* (RARβ), blocks amyloidogenic enzyme activity—forming a dual-track regulation of Aβ production (Radak et al., 2020). Notably, SIRT1 activation is induced by both aerobic and resistance exercise, indicating a broad role in exercise-mediated neuroprotection.

#### 3.1.5 Glymphatic clearance

The glymphatic system is a cerebrospinal fluid (CSF)–interstitial fluid (ISF) exchange pathway dependent on astrocytic aquaporin 4 (AQP4). Pulsatile forces and astrocyte endfeet mediate CSF influx into brain parenchyma, clearing metabolic waste during sleep and rest (Iliff et al., 2012; Olegário et al., 2024). In AD, AQP4 polarity loss significantly weakens glymphatic clearance, exacerbating Aβ accumulation; Aerobic exercise can restore AQP4 polarization, enhance CSF–ISF exchange, and markedly reduce soluble Aβ levels in AD rodent brains, thereby delaying cognitive decline (Xie et al., 2013; Liu et al., 2022). Studies have shown that, in APP/PS1 mice, moderate aerobic exercise improved glymphatic efficiency, accompanied by upregulated perivascular AQP4 expression and enhanced arterial pulsatility, thereby reducing brain soluble Aβ (Liang et al., 2025). Voluntary wheel running significantly accelerated glymphatic flow, promoting Aβ efflux via enhanced CSF–ISF exchange, while improving astrocytic AQP4 expression and polarization, reducing Aβ deposition and neuroinflammation (He et al., 2017).

#### 3.1.6 Microglial phenotype modulation

Microglia are the resident immune cells of the central nervous system, able to polarize between pro-inflammatory M1 and anti-inflammatory M2 states to regulate neuroinflammation and phagocytic clearance of pathological debris. They are crucial modulators in AD progression (Cherry et al., 2014). M1 microglia releases inflammatory cytokines, promoting neurotoxicity, whereas M2 microglia have phagocytic, tissue-repair, and neuroprotective functions. The M1/M2 balance determines brain inflammation levels and pathological clearance capacity—key factors in maintaining neural homeostasis and preventing AD (Lannes et al., 2017). Treadmill exercise can polarize hippocampal microglia in AD mice from an M1 to M2 phenotype; M2 microglia show significantly increased Arg1 and IL-10 expression, enhancing Aβ phagocytosis and degradation in the microenvironment (Liang et al., 2022).

#### 3.1.7 Exerkine signaling

Exerkines are exercise-induced, muscle-secreted bioactive molecules—such as FNDC5/irisin—that cross the blood–brain barrier to activate BDNF, PGC-1α, and other neuroplasticity and metabolic pathways in the brain. FNDC5/irisin levels in the hippocampus and cerebrospinal fluid of AD patients are significantly reduced; regular aerobic exercise, via PGC-1α upregulation, elevates FNDC5 expression, enhancing irisin-mediated neprilysin (NEP) secretion and ADAM10 activity, synergistically inhibiting BACE1 and accelerating Aβ degradation (Lourenco et al., 2019). *In vitro* and in mouse experiments, irisin also promotes astrocytic NEP secretion via ERK–STAT3 signaling, accelerating Aβ clearance, further corroborating the dual regulatory role of exercise factors in Aβ homeostasis (Kim et al., 2025) (please refer to Table 1 for detailed information).

**Table 1:** Major molecular pathways by which exercise promotes Aβ clearance.

Pathway	Exercise modality	Study population/model	Key molecular cascade	References (author, year)
Autophagy–lysosome pathway	Aerobic (treadmill; voluntary wheel)	APP/PS1 mice; aged mice	Exercise→ AMPK↑ → mTOR↓ → ULK1 activation→ Autophagosome formation → Fusion with lysosomes → Cathepsin D-mediated Tau/Aβ degradation	Zhao et al. (2018) and Jian et al. (2022)
PGC-1α → FNDC5 → BDNF cascade	Aerobic (moderate continuous); HIIT (where tested)	Aβ-induced rats; APP/PS1 mice	Exercise→AMPK↑ → PGC-1α↑ → FNDC5/irisin↑ → BDNF↑ → ADAM10↑& BACE1↓ → Reduced Aβ production	Azimi et al. (2018)
Lipid raft modulation	Aerobic (treadmill; intensity-stratified protocols)	APP/PS1 mice	Exercise→Reduced β/*γ*-secretase clustering in lipid rafts → Disruption of raft integrity → Relief of autophagy–lysosome blockade → Enhanced Aβ clearance	Zhang et al. (2019) and Zeng et al. (2020)
SIRT1-dependent pathway	Aerobic; resistance; combined (evidence across modalities)	Human skeletal muscle; rodents	Exercise → ↑NAD^+^ → SIRT1activation → Deacetylation →↑ADAM10&↑PGC-1α → RARβ-mediated BACE1 inhibition → Dual suppression of Aβ generation	Radak et al. (2020)
Glymphatic clearance pathway	Aerobic (wheel/treadmill); HIIT (select studies)	Aged mice; APP/PS1 mice; AQP4−/− mice (controls)	Exercise → Restoration of AQP4 polarity & increased vascular pulsatility → ↑CSF–ISF exchange → ↑Aβ efflux → Decreased soluble brain Aβ	He et al. (2017), Liu et al. (2022), and Liang et al. (2025)
Microglial phenotype shift (M1 → M2)	Aerobic (treadmill)	APP/PS1 mice	Exercise → ↑IL-4/IL-13–STAT6 & ↓NF-κB → M2 polarization → ↑Arg1, ↑IL-10 → Enhanced Aβ phagocytosis → Reduced neuroinflammation	Cherry et al. (2014) and Liang et al. (2022)
Exerkine-mediated signaling	Aerobic (wheel/treadmill); HIIT (reported in some models)	Rodent AD models with human/CSF corroboration	Exercise → ↑PGC-1α → ↑FNDC5/irisin → ↑ERK/STAT3 → ↑NEP & ↑ADAM10 → ↓BACE1 → Accelerated Aβ degradation	Lourenco et al. (2019) and Kim et al. (2025)

### 3.2 Exercise improves Tau protein load

Multiple animal studies (primarily using aerobic exercise) show that regular physical activity reduces Tau accumulation in the brain and ameliorates Tau-related pathology (Brown et al., 2019; Belarbi et al., 2011). Human research finds that individuals with higher physical activity levels have lower Tau burdens in brain tissue or blood (Desai et al., 2021; Brown et al., 2018). Among older adults with high total tau, medium physical activity was associated with a 58% slower rate of global cognitive decline and high physical activity with a 41% slower rate; among those with low total tau, the association was comparatively lower (Desai et al., 2021). Exercise may affect Tau expression and aggregation via multiple pathways, including modulation of neuronal activity, antioxidant capacity, and inflammatory responses (Belarbi et al., 2011; Daniele et al., 2017; Wu et al., 2016).

## 4 The suprachiasmatic nucleus and clock genes

The SCN is widely recognized as the master circadian pacemaker, controlling behavioral rhythms and coordinating peripheral clocks in organs such as the liver, kidney, and muscle (Song et al., 2015; Kumar et al., 2024). Through neural and humoral signals, the SCN synchronizes peripheral clocks, ensuring that systemic physiological processes align with the environmental light–dark cycle; conversely, peripheral clocks contribute to rhythm regulation via metabolites, gut hormones, and neural feedback to the SCN, forming a bidirectional coupling network that optimizes energy utilization and organismal function (Mohawk et al., 2012). Under normal conditions, this central–peripheral coordination ensures synchronous rhythmicity of behavior (e.g., sleep–wake), endocrine (e.g., melatonin, cortisol), and other physiological processes. When SCN function is impaired or peripheral clocks are desynchronized, global circadian disruption ensues, manifesting as sleep disorders, cognitive decline, and other pathological states (Reilly et al., 2007).

In mammals, circadian rhythms are regulated by a core molecular clock mechanism centered on a dynamic balance between transcriptional activators (CLOCK/NPAS2 and BMAL1) and repressors (mPER, mCRY), with CREB-binding protein (CBP) serving as a key coactivator that, together with BMAL1, drives downstream clock gene transcription. This molecular network operates within the SCN to regulate circadian output signals, maintaining synchronization of behavioral and physiological rhythms (Song et al., 2015). SCN deterioration can exacerbate circadian disruption in AD patients.

Exercise, as a non-photic zeitgeber, has been widely shown to modulate core and peripheral clock gene expression, thereby influencing sleep–wake rhythms. In human and animal models, both acute single bouts of exercise and chronic regular training significantly affect expression of key clock genes such as BMAL1 and PER2.

### 4.1 Amplitude amplification and rhythm stabilization

Long-term, time-locked aerobic or resistance training can re-entrain the central circadian pacemaker, stabilizing and strengthening clock-gene rhythms within the suprachiasmatic nucleus (SCN) by increasing the amplitude of core clock-gene oscillations and improving cycle-to-cycle stability of their peaks and troughs. In mice held in constant darkness, scheduled voluntary wheel running aligned SCN Per1-luc oscillations and preserved internal temporal order between the SCN and peripheral tissues, while simultaneously preventing the progressive damping of Per1-luc bioluminescence normally observed under constant conditions and maintaining high-amplitude oscillations across successive cycles—evidence that chronic exercise acts as a bona fide non-photic zeitgeber for the central molecular clock (Sato and Yamanaka, 2023). In complementary models, 3 weeks of timed daily running remodeled SCN network coupling by reducing GABA_A inhibition, increased synchrony of SCN Per1 and Per2 gene rhythms, and enhanced the population-level amplitude and coherence of these rhythms, restoring stable approximately 24 h behavioral rhythms in mice with impaired VIP–VPAC2 signaling, thereby functionally converting weak, low-amplitude SCN outputs into more robust, high-amplitude oscillations that are more resistant to internal and external perturbations (Hughes et al., 2021).

### 4.2 Phase adjustment and clock dependency

A single acute bout of exercise can transiently reset local clock gene phases. Hours after resistance exercise, muscle Per1 and Per2 mRNA peaks significantly advance; exercise timing determines phase shift direction—high-intensity evening exercise induces phase advances, potentially correcting “phase delay” type circadian disruption, whereas morning exercise induces phase delays, aiding in correcting “phase advance” misalignment. This time-of-day dependency provides a theoretical basis for personalized exercise prescriptions, making exercise an effective non-photic chronotherapeutic strategy (Aoyama and Shibata, 2020; Procopio and Esser, 2025).

### 4.3 Central–peripheral bidirectional coupling and indirect SCN influence

Although direct evidence of exercise’s effects on the SCN remains limited, and most available data are derived from a relatively small number of rodent studies and indirect human readouts, animal studies nevertheless suggest that physical activity can act as a non-photic zeitgeber that indirectly stabilizes the central clock via exercise-induced physiological cues. Such cues include changes in core body temperature, adrenergic activation, and neuroendocrine outputs such as glucocorticoids. Timed voluntary wheel running or scheduled treadmill exercise in rodents has been reported to reorganize molecular clock rhythms in the SCN and peripheral tissues and to restore more robust 24 h behavioral rhythms in models with impaired SCN neuropeptide signaling (Hitrec et al., 2023). In parallel, exercise exerts well-documented effects on peripheral clocks: time-restricted aerobic training alters Per1 and Bmal1 expression and the phase of cardiac and skeletal muscle rhythms, while chronic physical training in humans is associated with modified clock-gene expression in immune cells and a more anti-inflammatory profile (Schroeder et al., 2012; Dial et al., 2024). Beyond the exercise literature, broader circadian work indicates that peripheral metabolic and endocrine factors (e.g., leptin, ghrelin, FGF21, adiponectin, and inflammatory cytokines) can signal back to the hypothalamus and SCN via humoral and autonomic pathways, thereby modulating SCN clock-gene expression and neuronal activity and providing a mechanistic framework for central–peripheral bidirectional coupling (de Assis and Oster, 2021; Gachon et al., 2025; Dumbell et al., 2016). Together, these findings should therefore be viewed as preliminary and largely hypothesis-generating evidence that exercise-induced changes in peripheral clocks and inflammatory status may feed back onto the central circadian system via humoral and autonomic pathways, but this putative bidirectional coupling between peripheral oscillators and the SCN remains incompletely validated and will require targeted experimental testing before firm causal conclusions can be drawn.

## 5 Exercise-targeted modulation of melatonin

Melatonin, the principal marker of circadian phase shifts, is synthesized in the pineal gland from serotonin and its precursor tryptophan (Easton et al., 2024; Song, 2019). Its nocturnal rhythm under low-light conditions reliably reflects intrinsic timing and is closely tied to sleep propensity. During daylight, retinal photic signals travel via SCN–hypothalamus–spinal cord–superior cervical ganglia to the pineal gland, inhibiting norepinephrine release and suppressing melatonin synthesis. At night, this inhibitory pathway ceases, and melatonin peaks around midnight before declining at dawn. Thus, melatonin is an endogenous sleep “promoter,” used to treat insomnia and readjust circadian rhythms (Zisapel, 2018; Comai and Gobbi, 2024). With aging and certain diseases, melatonin production decreases or shifts (Homolak et al., 2018; Prodhan et al., 2021). In AD patients, nocturnal plasma and cerebrospinal melatonin levels are significantly lower, with peak secretion shifted early or late—reflecting phase misalignment (Nous et al., 2021; Steinbach and Denburg, 2024).

In early AD, reduced melatonin triggers sleep disturbances and circadian disruption, which further promotes Aβ accumulation; Aβ accumulation, in turn, inhibits melatonin production, forming a vicious cycle that exacerbates circadian disruption (Zhang et al., 2025; Cecon et al., 2015). Mechanistically, circadian or sleep loss elevates neuronal activity and impairs glymphatic clearance, increasing interstitial Aβ; in parallel, experimental work shows that soluble Aβ can directly impair pineal melatonin synthesis by activating NF-KB/ERK signaling in pinealocytes, downregulating the rate-limiting enzyme AANAT and thereby suppressing melatonin output (Kang et al., 2009; Xie et al., 2013; Zisapel, 2018; Cecon et al., 2015). Melatonin metabolite 6-sulfatoxymelatonin (aMT6s) excretion over 24 h is reduced in AD patients, with decreased nighttime peak amplitude—indicating intrinsic pineal dysfunction (Nous et al., 2021). The circadian amplitude of melatonin in AD patients is notably flattened, with reduced day–night differences, correlating with sleep fragmentation and sundowning (Lin et al., 2021). As AD progresses, pineal calcification and neuroinflammation further reduce melatonin receptor (MT1/MT2) expression, diminishing neural responsiveness to melatonin signals and disrupting rhythmic feedback loops (Roy et al., 2022; Steinbach and Denburg, 2024).

Whether exercise can effectively counteract melatonin dysregulation in AD patients remains debated; some studies report increased, decreased, or unchanged melatonin levels after exercise (Bian et al., 2025; Korkutata et al., 2025). From a mechanistic perspective, acute exercise often transiently elevates melatonin: increased exercise intensity activates the sympathetic nervous system, elevating plasma norepinephrine (NE) and epinephrine (EPI). NE binds *β*₁-adrenergic receptors on pinealocytes, stimulating G_s protein, which activates adenylyl cyclase to convert ATP into cyclic AMP (cAMP). Elevated cAMP activates protein kinase A (PKA), which phosphorylates and activates arylalkylamine N-acetyltransferase (AANAT)—the rate-limiting enzyme in melatonin synthesis—catalyzing serotonin to N-acetylserotonin, which is then methylated by hydroxyindole O-methyltransferase (HIOMT/ASMT) to form melatonin (Souissi et al., 2022). Thus, under consistent environmental conditions, a single moderate-to-high intensity exercise bout temporarily amplifies the NE–cAMP–PKA–AANAT cascade, leading to a short-term increase in nocturnal melatonin peak and overall secretion.

However, in practical research, there are often differences in experimental results due to various factors (Burgess and Fogg, 2008). The most powerful confounding factor is ambient light, light is the primary zeitgeber for the human circadian system and its presence, even at low levels, potently suppresses pineal melatonin synthesis. Many exercise studies, especially older or field-based trials, are conducted outdoors or in brightly-lit indoor gymnasiums. Light intensity is inherently unstable, changing based on time of day, cloud cover, and season. Even a slight variation in start time (e.g., 5:00 p.m. vs. 5:30 p.m.) can mean a dramatic difference in light exposure (lux), which can easily override, mask, or completely blunt any potential exercise-induced effect. This makes comparisons between studies, and even between subjects in the same study, exceptionally difficult (Phillips et al., 2019; Giménez et al., 2022). Second, genetic factors and individual chronotype create significant inter-subject variability. Genetic polymorphisms in core clock genes (e.g., PER2, BMAL1) or in the AANAT enzyme itself can alter an individual’s sensitivity to circadian resetting. An individual’s baseline chronotype (i.e., being a “lark” vs. an “owl”) will fundamentally change their response to the same exercise stimulus (Shen et al., 2023; Thomas et al., 2020). Finally, factors such as exercise type and duration also influence melatonin levels. However, these variables are relatively easier to control in experiments compared to environmental light exposure and individual genetic differences. Therefore, the divergent findings on exercise-induced melatonin changes are likely due to these confounding factors; under well-controlled conditions, exercise tends to reliably increase melatonin levels. For instance, a recent study of 80 healthy males aged 18–65 reported that after 12 weeks of HIIT, melatonin levels significantly increased, a finding made more persuasive by the rigorous control of light and sampling conditions (Al-Rawaf et al., 2023).

Exercise influences melatonin across four key dimensions: (1) phase shifting; (2) overall secretion changes; (3) amplitude enhancement; and (4) timing stability.

### 5.1 Phase shifting

Morning and forenoon exercise can “advance” the clock, shifting melatonin secretion earlier, facilitating earlier sleep onset, and alleviating circadian disruption; conversely, evening or pre-sleep high-intensity exercise can “delay” the clock, shifting melatonin secretion later, causing delayed sleep onset and exacerbating insomnia. One-hour moderate-intensity running (treadmill or outdoor) performed in the morning (~07:00 a.m.) or early afternoon (13:00–16:00) can induce significant phase advances, whereas evening (19:00–22:00) exercise leads to phase delays (Youngstedt et al., 2019). Youngstedt et al. established an exercise phase response curve (PRC) in 51 older and 48 younger healthy adults, showing that, similar to light, exercise at these times modulates clock phases of urinary aMT6s onset and peak, thereby advancing or delaying nocturnal melatonin release to correct phase-delayed or phase-advanced circadian disruption (Youngstedt et al., 2019). This finding provides a scientific basis for personalized exercise prescriptions in AD patients (e.g., morning exercise to advance rhythms; avoid high-intensity evening exercise to prevent delayed sleep).

Exercise modulates melatonin phase shifts by elevating SCN neuronal intracellular Ca^2+^, activating cAMP/PKA signaling, and phosphorylating CREB at Ser133, thereby upregulating Per1 and Per2 transcription to achieve phase advances or delays (Tahara et al., 2017; Korf and von Gall, 2024). Morning exercise–induced mild body temperature elevation also enhances HSF1 activity in the SCN, collaborating with CREB to promote Per gene expression and induce phase advances (Lee et al., 2024). Evening high-intensity exercise prolongs PCREB activation window in the SCN, delaying Per1/Per2 peak expression and producing phase delay effects. Additionally, peripheral cytokines (e.g., BDNF) induced by muscle exercise cross the blood–brain barrier to influence SCN TRKB receptors, further modulating downstream MAPK pathways and aiding clock gene restructuring (Zsuga et al., 2018; Cheng and Lee, 2022).

### 5.2 Total secretion changes

A single moderate-to-high intensity exercise session transiently suppresses melatonin (especially for nocturnal exercise), but as night falls, total melatonin secretion returns to baseline or slightly above. Boden et al. observed in 12 subjects that high-intensity treadmill running at night caused a transient spike in plasma melatonin (20–50% above resting levels), which returned to baseline within 30–60 min post-exercise; similar daytime (09:00–13:00) exercise induced mild melatonin increases, more pronounced under reduced light conditions (Buxton et al., 2003). Although this transient peak alone is insufficient to reshape rhythms, supplemental nocturnal melatonin may deepen subsequent slow-wave sleep.

During acute moderate-to-high intensity aerobic exercise, sympathetic-induced NE acts on pineal *β*₁-adrenergic receptors, activating AC–cAMP–PKA and promoting AANAT phosphorylation; however, exercise-induced cortisol and light factors transiently suppress AANAT gene transcription, lowering melatonin peak (Zemkova et al., 2011; Schomerus et al., 2003). After exercise, NE levels fall, PKA-mediated AANAT phosphorylation persists, and the CREB/CBP complex more readily binds the AANAT promoter, inducing mRNA rebound, forming a melatonin “rebound peak” (Schomerus et al., 2003). Simultaneously, exercise-released IL-6 and TNF-*α* downregulate RORα via STAT3 signaling, weakening its inhibition of AANAT, thereby further enhancing nocturnal melatonin secretion (Song, 2019; Moravcová et al., 2021).

### 5.3 Amplitude enhancement

Regular moderate-intensity aerobic exercise can amplify nocturnal melatonin peaks and lower daytime baselines, thereby enhancing circadian amplitude contrast, deepening slow-wave sleep, reducing nocturnal awakenings, and restoring sleep–wake rhythm stability and consistency, improving circadian disturbance (Tanaka et al., 2023). A systematic review found that compared to controls, long-term aerobic exercise increased aMT6s amplitude (acrophase) by 15–25% on average, lengthened nighttime peak duration, and flattened daytime troughs, significantly correlating with improved slow-wave sleep amount and sleep efficiency (Kim et al., 2023). In AD patients, such amplitude enhancement can strengthen nocturnal sleep signals and counteract fragmented sleep due to rhythm flattening. Buxton et al. observed that 60 min of nocturnal running increased melatonin peak by ~25% relative to baseline and further suppressed daytime baseline, creating a more pronounced day–night contrast. This amplitude enhancement is closely associated with increased deep sleep proportion and overall sleep quality (Buxton et al., 2003).

Long-term moderate-intensity aerobic exercise raises whole-body NAD^+^/NADH ratios, activating SIRT1 deacetylase, leading to deacetylation of BMAL1 and PER2, and enhancing CLOCK/BMAL1 dimer transcriptional activity on E-box elements of AANAT and ASMT genes—thereby amplifying nocturnal melatonin peaks and lowering daytime baselines (Chang and Guarente, 2013; Foteinou et al., 2018). Exercise-induced PGC-1α upregulation in the pineal gland binds directly to E-box sites on the AANAT promoter, synergizing with CLOCK/BMAL1 complexes to further expand day–night amplitude differences. SIRT1 deacetylation of REV-ERBα reduces its repression of Bmal1, resulting in higher nocturnal BMAL1 peaks and lower daytime baselines, strengthening melatonin circadian amplitude (Foteinou et al., 2018; Sweeney and Song, 2016; Fernández-Martínez et al., 2023).

### 5.4 Timing stabilization

Long-term endurance athletes often exhibit earlier melatonin onset and prolonged peak duration at night, maintaining stable nocturnal elevated melatonin levels. In a 6-week, moderate-intensity aerobic exercise randomized controlled trial among 40 sedentary adults, the exercise group showed a 25 min advance in dim-light melatonin onset (DLMO), an 18% increase in peak levels compared to baseline, and a 45 min extension of peak duration (Zhao et al., 2024). Another 8-week resistance training study in older adults reported a ~ 30 min increase in nighttime peak duration and 15% increase in total melatonin output, accompanied by a 28% reduction in nighttime awakenings (Kim et al., 2023). This “timing stabilization” not only optimizes continuous sleep–wake rhythm but also reduces nighttime awakenings—critical for AD patients, who experience frequent nocturnal arousals and difficulty initiating sleep; stable melatonin signals significantly improve sleep continuity and depth.

**Figure 1:**
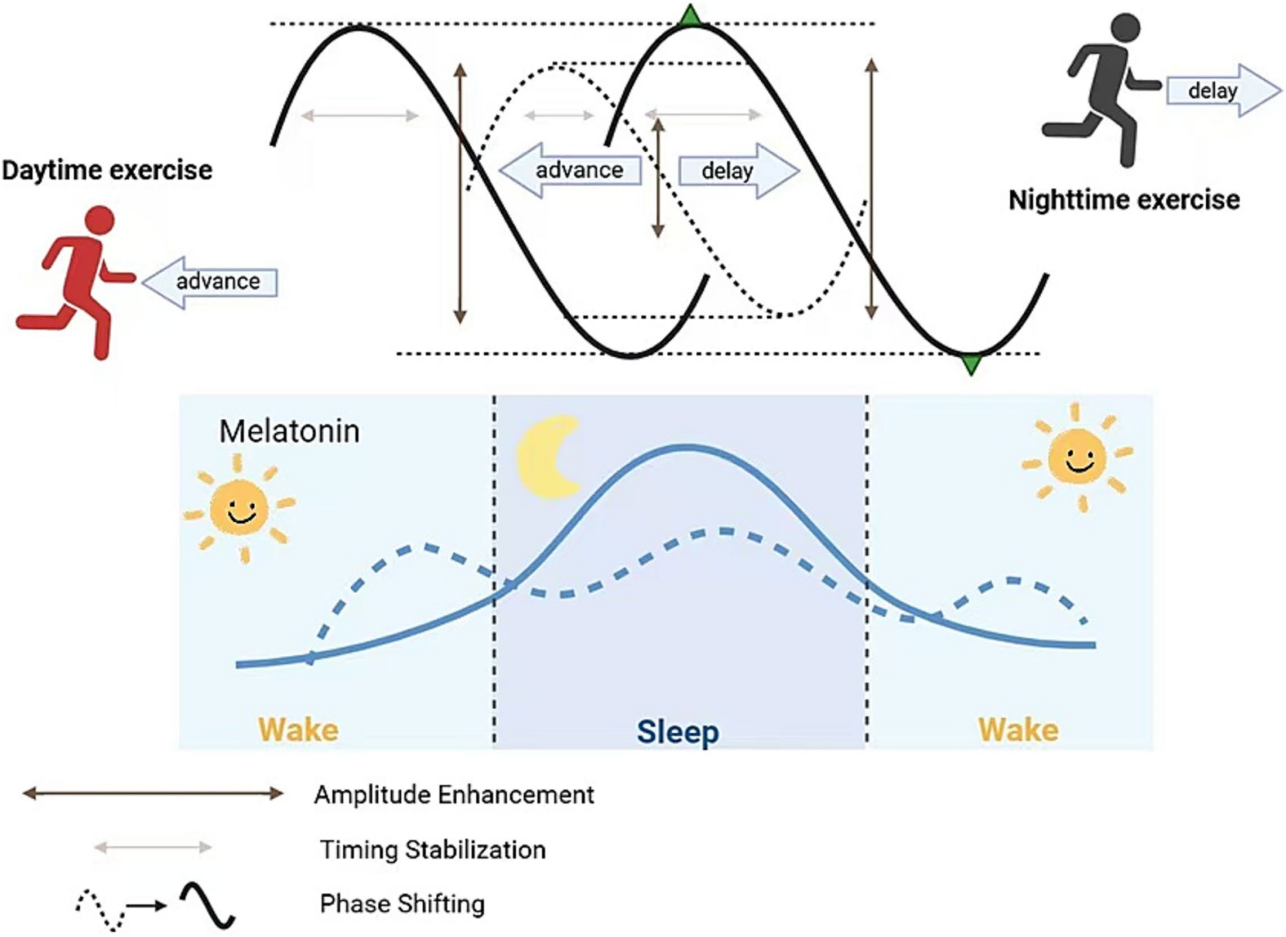
The effect of exercise on melatonin at different time periods.

Regular aerobic exercise enhances SCN VIP and PACAP neuropeptide secretion and synchrony. VIP, via VPAC₂ receptors, activates SCN cAMP/PKA/CREB signaling, advancing DLMO and prolonging peak duration (Hughes et al., 2021). Concurrently, exercise-activated AMPK in the pineal gland enhances CREB binding affinity to the AANAT promoter, advancing DLMO by ~20–30 min on average and extending peak duration (Barger et al., 2004). miR-132 has been shown to fine-tune circadian rhythms in the hypothalamus by targeting CLOCK and PER2 expression; although direct evidence of exercise-induced miR-132 effects on pineal PER2 and melatonin secretion is lacking, given that exercise can upregulate miR-132, it is speculated that similar mechanisms may modulate melatonin timing stability (please refer to Figure 1 for detailed information).

## 6 Conclusion

Exercise offers a multi-scale countermeasure against the circadian disruptions associated with AD pathology. At the molecular level, physical activity promotes Aβ clearance, inhibits tau aggregation, and reduces neuroinflammation via pathways involving BDNF, SIRT1, PGC-1α and various exercise-induced factors (exerkines). At the cellular level, exercise — acting as a non-photic zeitgeber — amplifies core clock gene oscillation (e.g., increases BMAL1–PER2 amplitude, shifts Per1/Per2 phase) while activating SCN neuropeptides (such as VIP and AVP) and reshaping peripheral clock-gene expression patterns. At the systemic level, it helps restore the amplitude and phase stability of the melatonin rhythm, thereby stabilizing the overall sleep–wake cycle. Importantly, these data support a unified Aβ/tau–clock–melatonin axis. Exercise-enhanced Aβ and tau clearance—via upregulated Aβ-degrading enzymes, improved glymphatic flux, and autophagy—diminishes their inhibitory effects on core clock components and SCN integrity. Normalized BMAL1/CLOCK oscillations, together with restored, high-amplitude melatonin rhythms, stabilize sleep–wake timing and deepen slow-wave sleep, further boosting glymphatic and proteostatic removal of neurotoxic species. Through this reciprocal reinforcement, appropriately timed physical activity has the potential to re-synchronize circadian organization and attenuate AD-related pathology in an integrated rather than pathway-by-pathway manner.

There is emerging human evidence supporting these benefits, although it remains limited. Preliminary clinical studies suggest that exercise protocols timed to an individual’s chronotype can yield improvements in sleep efficiency, daytime alertness, and short-term memory in older adults with cognitive impairment. However, these findings are tentative and come from small-scale trials with short durations and heterogeneous methods. In fact, much of our mechanistic understanding still stems from animal and cellular models; thus, caution is warranted when extrapolating to clinical practice. Larger, well-controlled human trials are needed to confirm that exercise-driven circadian improvements translate into tangible cognitive benefits for AD patients. Most current human studies lack statistical power and critical analyses because their sample sizes are typically well below 50 participants per arm. Assuming small-to-moderate effect sizes on sleep and cognitive outcomes (Cohen’s *d* ≈ 0.3–0.4), parallel-group randomized controlled trials with approximately 100–150 participants per group (total *N* ≈ 200–300) would be required to achieve 80–90% power at a two-sided *α* = 0.05, even after accounting for 20–25% attrition (Zhou et al., 2022). Future trials should therefore be designed at this scale or larger if the goal is to robustly detect clinically meaningful benefits of circadian-targeted exercise interventions in AD (Huang et al., 2017).

Translating these insights into standardized clinical practice also faces methodological challenges, particularly the heterogeneity in patient characteristics and intervention protocols. Differences in exercise modality, intensity, timing, and participants’ baseline circadian profiles complicate direct comparisons and generalizability. To address this, future randomized controlled trials should incorporate standardized circadian phenotyping and other design improvements to reduce variability. For example, using wearable actigraphy to track rest–activity patterns, measuring dim-light melatonin onset (DLMO) to determine each participant’s internal circadian phase, and profiling clock-gene expression rhythms in blood could allow researchers to stratify participants by circadian phenotype and tailor exercise timing to the individual (Cremascoli et al., 2021; Wittenbrink et al., 2018). Aligning exercise sessions relative to an individual’s internal clock (e.g., prescribing activity a certain number of hours before or after their DLMO) and adjusting analyses for each person’s baseline phase and amplitude may improve consistency of outcomes. Additionally, future trials should aim for longer intervention periods (beyond 12 weeks) to assess the durability of benefits and to observe any potential disease-modifying effects on AD progression.

Beyond clinical outcomes, mechanistic studies in humans are needed to validate key pathways suggested by preclinical research. For example, it remains to be verified whether exercise-induced upregulation of regulators like PGC-1α and SIRT1 actually occurs in the aging human brain, and whether peripheral exercise-derived factors (exerkines) released during workouts cross the blood–brain barrier in sufficient concentrations to trigger the neuroprotective signaling observed in animal models. Advanced neuroimaging techniques (such as PET scans with Aβ or tau tracers) could be employed in future trials to directly visualize whether exercise interventions slow or reverse AD pathology *in vivo*. Obtaining such mechanistic evidence in humans would solidify the causal link between restoring circadian rhythms through exercise and achieving neuroprotection in AD.

Finally, an important translational challenge is designing exercise programs that are feasible and safe for AD patients who often have significant mobility impairments or frailty, while still providing a sufficient physiological stimulus to preserve or improve physical function and circadian regulation. Many standard exercise regimens require standing balance and a degree of vigor that may be unfeasible for individuals with advanced age, arthritis, or high fall risk. Indeed, traditional standing workouts can be “difficult or impossible for those who are immobile or severely balance-impaired” (Efendi et al., 2023). Recent work in frail and cognitively impaired older adults further indicates that upright, weight-bearing programs are often associated with low adherence and heightened concern about falls in this population. By contrast, chair-based or seated routines offer a safe alternative that reduce postural demands, minimize loading of weight-bearing joints, and allow graded adjustment of range of motion, resistance, and tempo according to individual capacity. To accommodate limited mobility, future interventions should emphasize low-impact, accessible formats—such as chair-based exercises, gentle range-of-motion and flexibility training, and light aerobic or resistance exercises that can be performed with support (Park et al., 2020; Cordes et al., 2021). as these protocols have been shown in older adults with dementia or frailty to improve lower-limb strength, sit-to-stand performance, and functional mobility with a low incidence of adverse events. Implementing these regimens in supervised, therapist-guided programs (or with caregiver assistance) will help ensure participant safety and adherence and facilitate progressive titration of exercise volume and complexity over time. By tailoring the exercise mode and intensity to each patient’s functional abilities, even frail AD patients can be engaged in regular physical activity and potentially reap the circadian and cognitive benefits suggested by emerging chair-based intervention studies.

## References

[ref1] Al-Rawaf H. A. Gabr S. A. Iqbal A. Alghadir A. H. J. M. (2023). Effects of high-intensity interval training on melatonin function and cellular lymphocyte apoptosis in sedentary middle-aged men. Medicina 59:1201. doi: 10.3390/medicina59071201, 37512013 PMC10384261

[ref2] Aoyama S. Shibata S. (2020). Time-of-day-dependent physiological responses to meal and exercise. Front. Nutr. 7:18. doi: 10.3389/fnut.2020.00018 32181258 PMC7059348

[ref3] Auger R. R. Burgess H. J. Emens J. S. Deriy L. V. Thomas S. M. Sharkey K. M. (2015). Clinical practice guideline for the treatment of intrinsic circadian rhythm sleep-wake disorders: advanced sleep-wake phase disorder (aswpd), delayed sleep-wake phase disorder (dswpd), non-24-hour sleep-wake rhythm disorder (n24swd), and irregular sleep-wake rhythm disorder (iswrd). An update for 2015: an American Academy of sleep medicine clinical practice guideline. J. Clin. Sleep Med. 11, 1199–1236. doi: 10.5664/jcsm.5100, 26414986 PMC4582061

[ref4] Azimi M. Gharakhanlou R. Naghdi N. Khodadadi D. Heysieattalab S. (2018). Moderate treadmill exercise ameliorates amyloid-β-induced learning and memory impairment, possibly via increasing ampk activity and up-regulation of the pgc-1α/fndc5/bdnf pathway. Peptides 102, 78–88. doi: 10.1016/j.peptides.2017.12.027, 29309801

[ref5] Barger L. K. Wright K. P. Jr. Hughes R. J. Czeisler C. A. (2004). Daily exercise facilitates phase delays of circadian melatonin rhythm in very dim light. Am. J. Physiol. Regul. Integr. Comp. Physiol. 286, R1077–R1084. doi: 10.1152/ajpregu.00397.2003, 15031136

[ref6] Belarbi K. Burnouf S. Fernandez-Gomez F. J. Laurent C. Lestavel S. Figeac M. . (2011). Beneficial effects of exercise in a transgenic mouse model of alzheimer's disease-like tau pathology. Neurobiol. Dis. 43:486. doi: 10.1016/j.nbd.2011.04.022, 21569847

[ref7] Bian R. Xiang L. Su Z. (2025). Harnessing the benefits of physical exercise-induced melatonin: a potential promising approach to combat alzheimer's disease by targeting beta-amyloid (aβ). Hormones (Athens) 24, 3–13. doi: 10.1007/s42000-024-00602-6, 39312178

[ref8] Blazer D. G. Hays J. C. Foley D. J. (1995). Sleep complaints in older adults: a racial comparison. J. Gerontol. A Biol. Sci. Med. Sci. 50, M280–M284. doi: 10.1093/gerona/50a.5.m280, 7671031

[ref9] Brown B. Peiffer J. Rainey-Smith S. (2019). Exploring the relationship between physical activity, beta-amyloid and tau: a narrative review. Ageing Res. Rev. 50, 9–18. doi: 10.1016/j.arr.2019.01.003, 30615936

[ref10] Brown B. Rainey-Smith S. R. Dore V. Peiffer J. J. Burnham S. C. Laws S. M. . (2018). Self-reported physical activity is associated with tau burden measured by positron emission tomography. J. Alzheimers Dis. 63:1299. doi: 10.3233/JAD-170998, 29758940

[ref11] Burgess H. J. Fogg L. F. (2008). Individual differences in the amount and timing of salivary melatonin secretion. PLoS One 3:e3055. doi: 10.1371/journal.pone.0003055, 18725972 PMC2516604

[ref12] Buxton O. M. Lee C. W. L'Hermite-Baleriaux M. Turek F. W. Van Cauter E. (2003). Exercise elicits phase shifts and acute alterations of melatonin that vary with circadian phase. Am. J. Physiol. Regul. Integr. Comp. Physiol. 284, R714–R724. doi: 10.1152/ajpregu.00355.2002, 12571075

[ref13] Cecon E. Chen M. Marçola M. Fernandes P. A. C. Jockers R. Markus R. P. (2015). Amyloid β peptide directly impairs pineal gland melatonin synthesis and melatonin receptor signaling through the erk pathway. FASEB J. 29, 2566–2582. doi: 10.1096/fj.14-265678, 25757565

[ref14] Chang H. C. Guarente L. (2013). Sirt1 mediates central circadian control in the scn by a mechanism that decays with aging. Cell 153, 1448–1460. doi: 10.1016/j.cell.2013.05.027, 23791176 PMC3748806

[ref15] Cheng S.-M. Lee S.-D. (2022). Exercise training enhances BDNF/TrkB signaling pathway and inhibits apoptosis in diabetic cerebral cortex. Int. J. Mol. Sci. 23:6740. doi: 10.3390/ijms23126740, 35743182 PMC9223566

[ref16] Cherry J. D. Olschowka J. A. O'Banion M. K. (2014). Neuroinflammation and m2 microglia: the good, the bad, and the inflamed. J. Neuroinflammation 11:98. doi: 10.1186/1742-2094-11-98, 24889886 PMC4060849

[ref17] Comai S. Gobbi G. (2024). Melatonin, melatonin receptors and sleep: moving beyond traditional views. J. Pineal Res. 76:e13011. doi: 10.1111/jpi.13011, 39400423

[ref18] Cordes T. Schoene D. Kemmler W. Wollesen B. (2021). Chair-based exercise interventions for nursing home residents: a systematic review. J. Am. Med. Dir. Assoc. 22, 733–740. doi: 10.1016/j.jamda.2020.09.042, 33218912

[ref19] Cordy J. M. Hooper N. M. Turner A. J. (2006). The involvement of lipid rafts in Alzheimer's disease. Mol. Membr. Biol. 23, 111–122. doi: 10.1080/09687860500496417, 16611586

[ref20] Cremascoli R. Sparasci D. Giusti G. Cattaldo S. Prina E. Roveta F. . (2021). Effects of circadian phase tailored light therapy on sleep, mood, and cognition in Alzheimer's disease: preliminary findings in a pivotal study. Front. Physiol. 12:755322. doi: 10.3389/fphys.2021.755322, 35069234 PMC8770402

[ref21] Daniele S. Pietrobono D. Fusi J. Iofrida C. Chico L. Petrozzi L. . (2017). Α-synuclein aggregates with β-amyloid or tau in human red blood cells: correlation with antioxidant capability and physical exercise in human healthy subjects. Mol. Neurobiol. 55, 2653–2675. doi: 10.1007/s12035-017-0523-5, 28421539

[ref22] de Assis L. V. M. Oster H. (2021). The circadian clock and metabolic homeostasis: entangled networks. Cell. Mol. Life Sci. 78, 4563–4587. doi: 10.1007/s00018-021-03800-2, 33683376 PMC8195959

[ref23] Desai P. Evans D. Dhana K. Aggarwal N. T. Wilson R. S. McAninch E. . (2021). Longitudinal association of total tau concentrations and physical activity with cognitive decline in a population sample. JAMA Netw. Open 4:10.1001/jamanetworkopen.2021.20398. doi: 10.1001/jamanetworkopen.2021.20398, 34379124 PMC8358733

[ref24] Dial M. B. Malek E. M. Neblina G. A. Cooper A. R. Vaslieva N. I. Frommer R. . (2024). Effects of time-restricted exercise on activity rhythms and exercise-induced adaptations in the heart. Sci. Rep. 14:146. doi: 10.1038/s41598-023-50113-4, 38168503 PMC10761674

[ref25] Dumbell R. Matveeva O. Oster H. (2016). Circadian clocks, stress, and immunity. Front. Endocrinol. 7:37. doi: 10.3389/fendo.2016.00037, 27199894 PMC4852176

[ref26] Easton D. F. Gupta C. C. Vincent G. E. Ferguson S. A. J. C. B. (2024). Move the night way: how can physical activity facilitate adaptation to shift work? Commun. Biol. 7:259. doi: 10.1038/s42003-024-05962-8, 38431743 PMC10908783

[ref27] Eckhardt J. L. Isenberg L. Aslanyan V. Monreal T. Stradford J. Fenton L. . (2025). Circadian rhythms are associated with higher amyloid-β and tau and poorer cognition in older adults. Brain Commun. 7:fcaf322. doi: 10.1093/braincomms/fcaf322, 40926975 PMC12416565

[ref28] Efendi F. Tonapa S. I. Has E. M. M. Ho K. H. M. (2023). Effects of chair-based resistance band exercise on physical functioning, sleep quality, and depression of older adults in long-term care facilities: systematic review and meta-analysis. Int. J. Nurs. Sci. 10, 72–81. doi: 10.1016/j.ijnss.2022.12.002, 36860706 PMC9969069

[ref29] Fernández-Martínez J. Ramírez-Casas Y. Yang Y. Aranda-Martínez P. Martínez-Ruiz L. Escames G. . (2023). From chronodisruption to sarcopenia: the therapeutic potential of melatonin. Biomolecules 13:1779. doi: 10.3390/biom13121779, 38136651 PMC10741491

[ref30] Foteinou P. T. Venkataraman A. Francey L. J. Anafi R. C. Hogenesch J. B. Doyle F. J. (2018). Computational and experimental insights into the circadian effects of Sirt1. Proc. Natl. Acad. Sci. USA 115, 11643–11648. doi: 10.1073/pnas.1803410115, 30348778 PMC6233098

[ref31] Gachon F. Bugianesi E. Castelnuovo G. Oster H. Pendergast J. S. Montagnese S. (2025). Potential bidirectional communication between the liver and the central circadian clock in MASLD. NPJ Metab. Health Dis. 3:15. doi: 10.1038/s44324-025-00058-1, 40225783 PMC11981938

[ref32] Giménez M. C. Stefani O. Cajochen C. Lang D. Deuring G. Schlangen L. J. M. (2022). Predicting melatonin suppression by light in humans: unifying photoreceptor-based equivalent daylight illuminances, spectral composition, timing and duration of light exposure. J. Pineal Res. 72:e12786. doi: 10.1111/jpi.12786, 34981572 PMC9285453

[ref33] Han S. M. Jang Y. J. Kim E. Y. Park S. A. (2022). The change in circadian rhythms in p301s transgenic mice is linked to variability in hsp70-related tau disaggregation. Exp. Neurobiol. 31, 196–207. doi: 10.5607/en22019, 35786641 PMC9272121

[ref34] He X. F. Liu D. X. Zhang Q. Liang F. Y. Dai G. Y. Zeng J. S. . (2017). Voluntary exercise promotes glymphatic clearance of amyloid beta and reduces the activation of astrocytes and microglia in aged mice. Front. Mol. Neurosci. 10:144. doi: 10.3389/fnmol.2017.00144, 28579942 PMC5437122

[ref35] Hitrec T. Petit C. Cryer E. Muir C. Tal N. Fustin J. M. . (2023). Timed exercise stabilizes behavioral rhythms but not molecular programs in the brain's suprachiasmatic clock. iScience 26:106002. doi: 10.1016/j.isci.2023.106002, 36866044 PMC9971895

[ref36] Holth J. K. Fritschi S. K. Wang C. Pedersen N. P. Cirrito J. R. Mahan T. E. . (2019). The sleep-wake cycle regulates brain interstitial fluid tau in mice and csf tau in humans. Science (New York, N.Y.) 363, 880–884. doi: 10.1126/science.aav2546, 30679382 PMC6410369

[ref37] Homolak J. Mudrovčić M. Vukić B. Toljan K. (2018). Circadian rhythm and Alzheimer’s disease. Med. Sci. 6:52. doi: 10.3390/medsci6030052, 29933646 PMC6164904

[ref38] Hu Y. Niu L. Chen Y. Yang H. Qiu X. Jiang F. . (2025). Voluntary wheel running exercise improves sleep disorder, circadian rhythm disturbance, and neuropathology in an animal model of alzheimer's disease. Alzheimers Dement. 21:e70314. doi: 10.1002/alz.70314, 40556345 PMC12187974

[ref39] Huang Z. Muniz-Terrera G. Tom B. D. M. (2017). Power analysis to detect treatment effects in longitudinal clinical trials for Alzheimer's disease. Alzheimers Dement. (NY) 3, 360–366. doi: 10.1016/j.trci.2017.04.007, 28890916 PMC5590710

[ref40] Hughes A. T. L. Samuels R. E. Baño-Otálora B. Belle M. D. C. Wegner S. Guilding C. . (2021). Timed daily exercise remodels circadian rhythms in mice. Commun. Biol. 4:761. doi: 10.1038/s42003-021-02239-2, 34145388 PMC8213798

[ref41] Iliff J. J. Wang M. Liao Y. Plogg B. A. Peng W. Gundersen G. A. . (2012). A paravascular pathway facilitates csf flow through the brain parenchyma and the clearance of interstitial solutes, including amyloid β. Sci. Transl. Med. 4:147ra111. doi: 10.1126/scitranslmed.3003748, 22896675 PMC3551275

[ref42] Jian Y. Yuan S. Yang J. Lei Y. Li X. Liu W. (2022). Aerobic exercise alleviates abnormal autophagy in brain cells of app/ps1 mice by upregulating adipor1 levels. Int. J. Mol. Sci. 23:10.3390/ijms23179921. doi: 10.3390/ijms23179921, 36077318 PMC9456508

[ref43] Kang J. E. Lim M. M. Bateman R. J. Lee J. J. Smyth L. P. Cirrito J. R. . (2009). Amyloid-beta dynamics are regulated by orexin and the sleep-wake cycle. Science (New York, N.Y.) 326, 1005–1007. doi: 10.1126/science.1180962, 19779148 PMC2789838

[ref44] Katsuki F. Gerashchenko D. Brown R. E. (2022). Alterations of sleep oscillations in alzheimer's disease: a potential role for gabaergic neurons in the cortex, hippocampus, and thalamus. Brain Res. Bull. 187:181. doi: 10.1016/j.brainresbull.2022.07.002, 35850189 PMC9563641

[ref45] Kim N. Ka S. Park J. (2023). Effects of exercise timing and intensity on physiological circadian rhythm and sleep quality: a systematic review. Phys. Act. Nutr. 27, 052–063. doi: 10.20463/pan.2023.0029, 37946447 PMC10636512

[ref46] Kim E. Tanzi R. E. Choi S. H. (2025). Therapeutic potential of exercise-hormone irisin in alzheimer's disease. Neural Regen. Res. 20, 1555–1564. doi: 10.4103/nrr.Nrr-d-24-00098, 38993140 PMC11688551

[ref47] Ko C. H. Takahashi J. S. (2006). Molecular components of the mammalian circadian clock. Hum. Mol. Genet. 15, R271–R277. doi: 10.1093/hmg/ddl207 16987893

[ref48] Kondratova A. A. Kondratov R. V. (2012). The circadian clock and pathology of the ageing brain. Nat. Rev. Neurosci. 13, 325–335. doi: 10.1038/nrn3208, 22395806 PMC3718301

[ref49] Korf H. W. von Gall C. J. (2024). Mouse models in circadian rhythm and melatonin research. J. Pineal Res. 76:e12986. doi: 10.1111/jpi.12986, 38965880

[ref50] Korkutata A. Korkutata M. Lazarus M. (2025). The impact of exercise on sleep and sleep disorders. NPJ Biol. Timing Sleep 2:5. doi: 10.1038/s44323-024-00018-w, 41290929

[ref51] Kumar A. Vaca-Dempere M. Mortimer T. Deryagin O. Smith J. G. Petrus P. . (2024). Brain-muscle communication prevents muscle aging by maintaining daily physiology. Science (New York, N.Y.) 384, 563–572. doi: 10.1126/science.adj8533, 38696572

[ref52] Lannes N. Eppler E. Etemad S. Yotovski P. Filgueira L. J. O. (2017). Microglia at center stage: a comprehensive review about the versatile and unique residential macrophages of the central nervous system. Oncotarget 8, 114393–114413. doi: 10.18632/oncotarget.23106, 29371994 PMC5768411

[ref53] Lee K. Hong K.-S. Park J. Park W. J. P. a (2024). Readjustment of circadian clocks by exercise intervention is a potential therapeutic target for sleep disorders: a narrative review. Phys. Act. Nutr. 28:35. doi: 10.20463/pan.2024.0014, 39097996 PMC11298283

[ref54] Liang S. Liu H. Wang X. Lin H. Zheng L. Zhang Y. . (2025). Aerobic exercise improves clearance of amyloid-β via the glymphatic system in a mouse model of alzheimer's disease. Brain Res. Bull. 222:111263. doi: 10.1016/j.brainresbull.2025.111263, 39971255

[ref55] Liang F. Sun F. He B. Wang J. (2022). Treadmill exercise promotes microglial β-amyloid clearance and prevents cognitive decline in app/ps1 mice. Neuroscience 491:122. doi: 10.1016/j.neuroscience.2022.03.043, 35398179

[ref56] Lin C. H. Chiu C. C. Lane H. Y. (2021). Trough melatonin levels differ between early and late phases of alzheimer disease. Clin. Psychopharmacol. Neurosci. 19, 135–144. doi: 10.9758/cpn.2021.19.1.135, 33508797 PMC7851471

[ref57] Liu Y. Hu P. P. Zhai S. Feng W. X. Zhang R. Li Q. . (2022). Aquaporin 4 deficiency eliminates the beneficial effects of voluntary exercise in a mouse model of alzheimer's disease. Neural Regen. Res. 17, 2079–2088. doi: 10.4103/1673-5374.335169, 35142700 PMC8848602

[ref58] Lourenco M. V. Frozza R. L. de Freitas G. B. Zhang H. Kincheski G. C. Ribeiro F. C. . (2019). Exercise-linked FNDC5/irisin rescues synaptic plasticity and memory defects in Alzheimer’s models. Nat. Med. 25, 165–175. doi: 10.1038/s41591-018-0275-4, 30617325 PMC6327967

[ref59] Lucey B. P. Hicks T. J. McLeland J. S. Toedebusch C. D. Boyd J. Elbert D. L. . (2018). Effect of sleep on overnight cerebrospinal fluid amyloid β kinetics. Ann. Neurol. 83, 197–204. doi: 10.1002/ana.25117, 29220873 PMC5876097

[ref60] Meyer N. Harvey A. G. Lockley S. W. Dijk D.-J. (2022). Circadian rhythms and disorders of the timing of sleep. Lancet 400, 1061–1078. doi: 10.1016/S0140-6736(22)00877-7, 36115370

[ref61] Mohawk J. A. Green C. B. Takahashi J. S. (2012). Central and peripheral circadian clocks in mammals. Annu. Rev. Neurosci. 35:445. doi: 10.1146/annurev-neuro-060909-153128, 22483041 PMC3710582

[ref62] Moravcová S. Filipovská E. Spišská V. Svobodová I. Novotný J. Bendová Z. (2021). The circadian rhythms of stat3 in the rat pineal gland and its involvement in arylalkylamine-n-acetyltransferase regulation. Life (Basel) 11:1105. doi: 10.3390/life11101105, 34685476 PMC8541109

[ref63] Nigam S. M. Xu S. Kritikou J. S. Marosi K. Brodin L. Mattson M. P. (2017). Exercise and BDNF reduce aβ production by enhancing α‐secretase processing of APP. J. Neurochem. 142, 286–296. doi: 10.1111/jnc.14034, 28382744 PMC5498234

[ref64] Nixon R. A. Rubinsztein D. C. (2024). Mechanisms of autophagy-lysosome dysfunction in neurodegenerative diseases. Nat. Rev. Mol. Cell Biol. 25, 926–946. doi: 10.1038/s41580-024-00757-5, 39107446 PMC12239022

[ref65] Nous A. Engelborghs S. Smolders I. (2021). Melatonin levels in the alzheimer's disease continuum: a systematic review. Alzheimer's Res Ther 13:52. doi: 10.1186/s13195-021-00788-6, 33622399 PMC7903801

[ref66] Olegário R. L. Nóbrega O. T. Camargos E. F. J. (2024). The newly discovered glymphatic system: the missing link between physical exercise and brain health? Front. Integr. Neurosci. 18:1349563. doi: 10.3389/fnint.2024.1349563, 38690084 PMC11058641

[ref67] Park J. Tolea M. I. Sherman D. Rosenfeld A. Arcay V. Lopes Y. . (2020). Feasibility of conducting nonpharmacological interventions to manage dementia symptoms in community-dwelling older adults: a cluster randomized controlled trial. Am. J. Alzheimers Dis. Other Dement. 35:1533317519872635. doi: 10.1177/1533317519872635, 31533443 PMC10623920

[ref68] Phillips A. J. K. Vidafar P. Burns A. C. McGlashan E. M. Anderson C. Rajaratnam S. M. W. . (2019). High sensitivity and interindividual variability in the response of the human circadian system to evening light. Proc. Natl. Acad. Sci. USA 116, 12019–12024. doi: 10.1073/pnas.1901824116, 31138694 PMC6575863

[ref69] Procopio S. B. Esser K. A. (2025). Clockwork conditioning: aligning the skeletal muscle clock with time-of-day exercise for cardiometabolic health. J. Mol. Cell. Cardiol. 198, 36–44. doi: 10.1016/j.yjmcc.2024.11.011, 39615287 PMC11780665

[ref70] Prodhan A. S. U. Prodhan A. H. M. S. U. Cavestro C. Kamal M. A. Islam M. A. (2021). Melatonin and sleep disturbances in Alzheimer’s disease. CNS Neurol. Disord. Drug Targets 20, 736–754. doi: 10.2174/1871527320666210804155617, 34348635

[ref71] Radak Z. Suzuki K. Posa A. Petrovszky Z. Koltai E. Boldogh I. (2020). The systemic role of sirt1 in exercise mediated adaptation. Redox Biol. 35:101467. doi: 10.1016/j.redox.2020.101467, 32086007 PMC7284913

[ref72] Reilly D. F. Westgate E. J. FitzGerald G. A. (2007). Peripheral circadian clocks in the vasculature. Arterioscler. Thromb. Vasc. Biol. 27, 1694–1705. doi: 10.1161/ATVBAHA.107.144923, 17541024

[ref73] Roh J. H. Huang Y. Bero A. W. Kasten T. Stewart F. R. Bateman R. J. . (2012). Disruption of the sleep-wake cycle and diurnal fluctuation of β-amyloid in mice with Alzheimer’s disease pathology. Sci. Transl. Med. 4:150ra122-150ra122. doi: 10.1126/scitranslmed.3004291, 22956200 PMC3654377

[ref74] Roy J. Wong K. Y. Aquili L. Uddin M. S. Heng B. C. Tipoe G. L. . (2022). Role of melatonin in alzheimer’s disease: from preclinical studies to novel melatonin-based therapies. Front. Neuroendocrinol. 65:100986. doi: 10.1016/j.yfrne.2022.100986, 35167824

[ref75] Sato R. Y. Yamanaka Y. (2023). Nonphotic entrainment of central and peripheral circadian clocks in mice by scheduled voluntary exercise under constant darkness. Am. J. Physiol. Regul. Integr. Comp. Physiol. 324, R526–r535. doi: 10.1152/ajpregu.00320.2022, 36802951

[ref76] Scheltens P. de Strooper B. Kivipelto M. Holstege H. Chételat G. Teunissen C. E. . (2021). Alzheimer's disease. Lancet 397, 1577–1590. doi: 10.1016/S0140-6736(20)32205-4, 33667416 PMC8354300

[ref77] Schomerus C. Laedtke E. Korf H. W. (2003). Norepinephrine-dependent phosphorylation of the transcription factor cyclic adenosine monophosphate responsive element-binding protein in bovine pinealocytes. J. Pineal Res. 34, 103–109. doi: 10.1034/j.1600-079x.2003.00011.x, 12562501

[ref78] Schroeder A. M. Truong D. Loh D. H. Jordan M. C. Roos K. P. Colwell C. S. (2012). Voluntary scheduled exercise alters diurnal rhythms of behaviour, physiology and gene expression in wild-type and vasoactive intestinal peptide-deficient mice. J. Physiol. 590, 6213–6226. doi: 10.1113/jphysiol.2012.233676, 22988135 PMC3530127

[ref79] Shearman L. P. Sriram S. Weaver D. R. Maywood E. S. Chaves I. Zheng B. . (2000). Interacting molecular loops in the mammalian circadian clock. Science 288, 1013–1019. doi: 10.1126/science.288.5468.1013, 10807566

[ref80] Shen B. Ma C. Wu G. Liu H. Chen L. Yang G. (2023). Effects of exercise on circadian rhythms in humans. Front. Pharmacol. 14:1282357. doi: 10.3389/fphar.2023.1282357, 37886134 PMC10598774

[ref81] Son G. Neylan T. C. Grinberg L. T. (2024). Neuronal and glial vulnerability of the suprachiasmatic nucleus in tauopathies: evidence from human studies and animal models. Mol. Neurodegener. 19:4. doi: 10.1186/s13024-023-00695-4, 38195580 PMC10777507

[ref82] Song J. (2019). Pineal gland dysfunction in alzheimer's disease: relationship with the immune-pineal axis, sleep disturbance, and neurogenesis. Mol. Neurodegener. 14:28. doi: 10.1186/s13024-019-0330-8, 31296240 PMC6624939

[ref83] Song H. Moon M. Choe H. K. Han D. H. Jang C. Kim A. . (2015). Aβ-induced degradation of bmal1 and cbp leads to circadian rhythm disruption in Alzheimer’s disease. Mol. Neurodegener. 10:13. doi: 10.1186/s13024-015-0007-x, 25888034 PMC4404698

[ref84] Souissi A. Dergaa I. Chtourou H. Ben Saad H. (2022). The effect of daytime ingestion of melatonin on thyroid hormones responses to acute submaximal exercise in healthy active males: a pilot study. Am. J. Mens Health 16:15579883211070383. doi: 10.1177/15579883211070383 35060417 PMC8785310

[ref85] Steinbach M. J. Denburg N. L. (2024). Melatonin in alzheimer’s disease: literature review and therapeutic trials. J Alzheimer's Dis 101, S193–S204. doi: 10.3233/JAD-230760, 39422936 PMC12633715

[ref86] Sun S.-Y. Chen G.-H. (2022). Treatment of circadian rhythm sleep–wake disorders. Curr. Neuropharmacol. 20, 1022–1034. doi: 10.2174/1570159x19666210907122933, 34493186 PMC9886819

[ref87] Sweeney G. Song J. (2016). The association between pgc-1α and alzheimer's disease. Anat. Cell Biol. 49, 1–6. doi: 10.5115/acb.2016.49.1.1, 27051562 PMC4819073

[ref88] Tahara Y. Aoyama S. Shibata S. J. (2017). The mammalian circadian clock and its entrainment by stress and exercise. J. Physiol. Sci. 67, 1–10. doi: 10.1007/s12576-016-0450-7, 27084533 PMC5138246

[ref89] Tan Z.-X. Dong F. Wu L.-Y. Feng Y.-S. Zhang F. J. M. N. (2021). The beneficial role of exercise on treating Alzheimer’s disease by inhibiting β-amyloid peptide. Mol. Neurobiol. 58, 5890–5906. doi: 10.1007/s12035-021-02514-7, 34415486

[ref90] Tanaka Y. Sagayama H. Shimizu K. J. C. N. O. S. (2023). Insights into exercise timing to regulate circadian clocks and phenotypes. Clin. Nutr. Open Sci. 47, 96–101. doi: 10.1016/j.nutos.2022.12.007

[ref91] Thomas J. M. Kern P. A. Bush H. M. McQuerry K. J. Black W. S. Clasey J. L. . (2020). Circadian rhythm phase shifts caused by timed exercise vary with chronotype. JCI Insight 5:10.1172/jci.insight.134270. doi: 10.1172/jci.insight.134270, 31895695 PMC7098792

[ref92] Van Erum J. Van Dam D. De Deyn P. P. (2018). Sleep and Alzheimer's disease: a pivotal role for the suprachiasmatic nucleus. Sleep Med. Rev. 40, 17–27. doi: 10.1016/j.smrv.2017.07.005, 29102282

[ref93] van Hattem T. Verkaar L. T. K. M. Krugliakova E. Adelhöfer N. Zeising M. Drinkenburg W. H. I. M. . (2024). Targeting sleep physiology to modulate glymphatic brain clearance. Physiology (Bethesda). doi: 10.1152/physiol.00019.2024 39601891

[ref94] Van Someren E. J. (2000). Circadian and sleep disturbances in the elderly. Exp. Gerontol. 35, 1229–1237. doi: 10.1016/s0531-5565(00)00191-1, 11113604

[ref95] Wang C. Holtzman D. M. (2020). Bidirectional relationship between sleep and alzheimer's disease: role of amyloid, tau, and other factors. Neuropsychopharmacology 45, 104–120. doi: 10.1038/s41386-019-0478-5, 31408876 PMC6879647

[ref96] Winer J. R. Deters K. D. Kennedy G. Jin M. Goldstein-Piekarski A. Poston K. L. . (2021). Association of short and long sleep duration with amyloid-β burden and cognition in aging. JAMA Neurol. 78, 1187–1196. doi: 10.1001/jamaneurol.2021.2876, 34459862 PMC8406215

[ref97] Wittenbrink N. Ananthasubramaniam B. Münch M. Koller B. Maier B. Weschke C. . (2018). High-accuracy determination of internal circadian time from a single blood sample. J. Clin. Invest. 128, 3826–3839. doi: 10.1172/jci120874, 29953415 PMC6118629

[ref98] Wu J. Wu J. W. Hussaini S. A. Bastille I. M. Rodriguez G. A. Mrejeru A. . (2016). Neuronal activity enhances tau propagation and tau pathology in vivo. Nat. Neurosci. 19, 1085–1092. doi: 10.1038/nn.4328, 27322420 PMC4961585

[ref99] Xie L. Kang H. Xu Q. Chen M. J. Liao Y. Thiyagarajan M. . (2013). Sleep drives metabolite clearance from the adult brain. Science (New York, N.Y.) 342, 373–377. doi: 10.1126/science.1241224, 24136970 PMC3880190

[ref100] Yang H. Niu L. Tian L. Hu Y. Cheng C. Li S. . (2025). Circadian rhythm disturbances in Alzheimer’s disease: insights from plaque-free and plaque-burdened stages in appswe/ps1de9 mice. Alzheimer's Res Ther 17:76. doi: 10.1186/s13195-025-01724-8, 40188157 PMC11971749

[ref101] Youngstedt S. D. Elliott J. A. Kripke D. F. (2019). Human circadian phase-response curves for exercise. J. Physiol. 597, 2253–2268. doi: 10.1113/jp276943, 30784068 PMC6462487

[ref102] Zemkova H. Stojilkovic S. S. Klein D. C. (2011). Norepinephrine causes a biphasic change in mammalian pinealocye membrane potential: role of alpha1b-adrenoreceptors, phospholipase c, and ca2+. Endocrinology 152, 3842–3851. doi: 10.1210/en.2011-1180, 21828176 PMC3176642

[ref103] Zeng B. Zhao G. Liu H. L. (2020). The differential effect of treadmill exercise intensity on hippocampal soluble aβ and lipid metabolism in APP/PS1 mice. Neuroscience 430, 73–81. doi: 10.1016/j.neuroscience.2020.01.005, 31954827

[ref104] Zhang X. Song W. J. A. (2013). The role of APP and BACE1 trafficking in APP processing and amyloid-β generation. Alzheimer's Res Ther 5:46. doi: 10.1186/alzrt211, 24103387 PMC3978418

[ref105] Zhang Z. Xue P. Bendlin B. B. Zetterberg H. de Felice F. Tan X. . (2025). Melatonin: a potential nighttime guardian against alzheimer's. Mol. Psychiatry 30, 237–250. doi: 10.1038/s41380-024-02691-6, 39128995 PMC11649572

[ref106] Zhang X. L. Zhao N. Xu B. Chen X. H. Li T. J. (2019). Treadmill exercise inhibits amyloid-β generation in the hippocampus of app/ps1 transgenic mice by reducing cholesterol-mediated lipid raft formation. Neuroreport 30, 498–503. doi: 10.1097/wnr.0000000000001230, 30882716

[ref107] Zhao Y. Dai Q. Li Y. Li C. (2024). Exercise therapy in the application of sleep disorders. Front. Neurol. 15:1324112. doi: 10.3389/fneur.2024.1324112, 38966079 PMC11222904

[ref108] Zhao N. Zhang X. Song C. Yang Y. He B. Xu B. (2018). The effects of treadmill exercise on autophagy in hippocampus of app/ps1 transgenic mice. Neuroreport 29, 819–825. doi: 10.1097/wnr.0000000000001038, 29672446 PMC5999367

[ref109] Zhou S. Chen S. Liu X. Zhang Y. Zhao M. Li W. (2022). Physical activity improves cognition and activities of daily living in adults with alzheimer's disease: a systematic review and meta-analysis of randomized controlled trials. Int. J. Environ. Res. Public Health 19:10.3390/ijerph19031216. doi: 10.3390/ijerph19031216, 35162238 PMC8834999

[ref110] Zisapel N. J. B. (2018). New perspectives on the role of melatonin in human sleep, circadian rhythms and their regulation. Br. J. Pharmacol. 175, 3190–3199. doi: 10.1111/bph.14116, 29318587 PMC6057895

[ref111] Zsuga J. More C. E. Erdei T. Papp C. Harsanyi S. Gesztelyi R. (2018). Blind spot for sedentarism: redefining the diseasome of physical inactivity in view of circadian system and the irisin/BDNF axis. Front. Neurol. 9:818. doi: 10.3389/fneur.2018.00818, 30333788 PMC6176117

